# Self‐Sensing Paper Actuators Based on Graphite–Carbon Nanotube Hybrid Films

**DOI:** 10.1002/advs.201800239

**Published:** 2018-05-16

**Authors:** Morteza Amjadi, Metin Sitti

**Affiliations:** ^1^ Physical Intelligence Department Max Planck Institute for Intelligent Systems 70569 Stuttgart Germany; ^2^ Max Planck‐ETH Center for Learning Systems Max Planck Institute for Intelligent Systems 70569 Stuttgart Germany

**Keywords:** carbon nanotubes, graphite, hybrid films, self‐sensing actuators, strain sensors

## Abstract

Soft actuators have demonstrated potential in a range of applications, including soft robotics, artificial muscles, and biomimetic devices. However, the majority of current soft actuators suffer from the lack of real‐time sensory feedback, prohibiting their effective sensing and multitask function. Here, a promising strategy is reported to design bilayer electrothermal actuators capable of simultaneous actuation and sensation (i.e., self‐sensing actuators), merely through two input electric terminals. Decoupled electrothermal stimulation and strain sensation is achieved by the optimal combination of graphite microparticles and carbon nanotubes (CNTs) in the form of hybrid films. By finely tuning the charge transport properties of hybrid films, the signal‐to‐noise ratio (SNR) of self‐sensing actuators is remarkably enhanced to over 66. As a result, self‐sensing actuators can actively track their displacement and distinguish the touch of soft and hard objects.

Soft actuator materials have received significant attention recently due to their importance in soft robotics, artificial muscles, biomimetic devices, and so forth.^1^, ^2^, ^3^ In recent years, new emerging functional materials, such as thermoresponsive polymers,^1^, ^4^, ^5^, ^6^, ^7^, ^8^ conducting polymers,^1^, ^9^, ^10^ paper,^1^, ^9^, ^11^, ^12^ dielectric elastomers,^13^, ^14^, ^15^ liquid‐crystal elastomers,^16^, ^17^, ^18^, ^19^ low‐dimensional carbons,^5^, ^7^, ^8^, ^12^, ^20^, ^21^, ^22^, ^23^, ^24^ and magnetic composite materials,^2^, ^25^ have considerably improved the deformation amplitude, response speed, force generation, and programmable motion output of soft actuators. Aside from efficient shape‐changing behavior of soft actuators, real‐time motion feedback is essential for their greater functionalities and wider adoption. To date, however, the actuation displacement is typically determined by bulky optical systems and image postprocessing, hindering effective and compact sensing capabilities of soft actuators.^1^, ^2^, ^11^, ^19^

Most recently, a few studies have demonstrated soft actuators with integrated mechanical‐sensing elements.^11^, ^20^, ^26^, ^27^, ^28^, ^29^ We have implemented flexible microcracks‐based strain sensors on pneumatically actuated soft gripper fingers to distinguish their touch status, contact force, and bending position.^26^ Phan et al. have reported a flexible actuator based on paper coated with ferromagnetic nanoparticles and graphite.^11^ Ferromagnetic nanoparticles were used to magnetically drive the actuator, while the actuation motion was detected by piezoresistive response of the graphite trace deposited on paper. In another work, stretchable optical waveguides have been utilized for strain sensing in a pneumatically actuated prosthetic hand.^29^ The curvature, elongation, and fingertip contact force of soft fingers were measured via innervated photonic sensors. All of these integrated devices, however, require multiple connection terminals and different energy inputs for synergistic actuation and sensation, still making the entire system complicated. Thus, it is highly desirable to simplify the integration of soft actuators and sensors, toward compact and multifunctional self‐sensing actuators.

Herein, we add sensing function to our recently reported multiresponsive actuators composed of normal copy paper and polypropylene (PP) film.^1^ The significance of this work is that the combination of functional materials overcomes the self‐sensing limitation of current soft actuators. We accomplish independent electrothermal stimulation and real‐time displacement sensation by the hybridization of graphite microparticles and CNTs. Given nearly zero thermal coefficient of resistance (TCR) and high piezoresistivity of hybrid films, the SNR of the proposed self‐sensing actuators is significantly boosted to 66.28, which is already 5.17 (129.96) times higher than the SNRs of actuators made of neat graphite (CNT) films. Importantly, high‐performance self‐sensing actuators can not only sense the self‐actuation sate but also recognize the touch of soft and hard objects. Additionally, they can track their dynamic motion upon other external stimuli like light irradiation.

**Figure** **1**a depicts the proposed structure of our bilayer actuators, which are composed of copy paper and PP film both as active layer materials. We utilized paper given its high coefficient of hygroscopic expansion (CHE) (CHE_Paper_ ≈ 0.1 C^−1^, C: moisture concentration of paper) plus a low coefficient of thermal expansion (CTE) (CTE_Paper_ ≈ 10 ppm K^−1^), and PP film due to its high CTE of about 137.5 ppm K^−1^ and negligible CHE.^1^ Indeed, bilayer actuators operate based on the large hygroscopic shrinkage of paper and simultaneously major thermal expansion of the PP film upon increasing their temperature.

**Figure 1:**
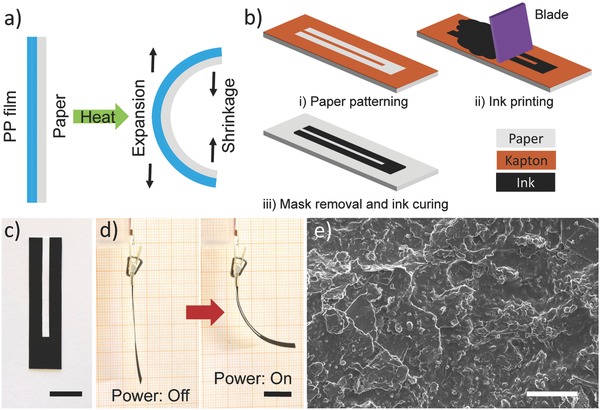
a) Schematic cross‐section of bilayer actuators made of paper and PP film. b) Fabrication processes of the electroresistive heater/sensory circuit on paper. c) Photograph of a U‐shaped conductive film patterned on paper (scale bar: 1 cm). d) Bending deformation of an actuator upon application of 30 V (scale bar: 1 cm). e) SEM image on the surface of the hybrid film coated on the paper substrate (scale bar: 40 µm).

After selecting active layer materials, electrically driven resistive heaters are directly deposited on paper to locally control the temperature of actuators. Figure 1b schematically illustrates the fabrication processes of the conductive circuit on paper (see the Experimental Section for details). Briefly, an A4 copy paper was first patterned by 110 µm thick Kapton tape in a U‐shaped geometry (Figures S1and S2, Supporting Information). A conductive ink made of graphite microparticles and CNTs was then uniformly screen printed on the patterned paper by means of a steel blade (see the Experimental Section for ink preparation). After removing Kapton tape, paper was put onto a hotplate under 100 °C for 30 min to solidify the deposited viscous ink. The post‐annealing of the coated paper at 150 °C for 30 min enabled a robust electroresistive heater on paper (Figure 1c). The assembly of bilayer actuators was finally completed by attaching the self‐adhesive PP film to the backside of the patterned paper (Figure 1d).

Scanning electron microscopy (SEM) images show the structural evolution from a bare paper to a conductive film‐coated paper (Figure S3, Supporting Information). The bare paper has a 3D porous network of intertwined cellulose fibers. After printing, the hybrid ink of graphite microparticles and CNTs evenly filled the gap among paper fibers and formed a conformal conductive layer on the surface of paper (Figure 1e). The fibrous structure and hydrophilicity of paper are particularly critical for the sufficient infiltration of the ink into the paper substrate (Figure S4, Supporting Information).^1^, ^9^ Additionally, it is known that both graphite and CNTs bind strongly to paper fibers.^30^, ^31^ As a result, no delamination or fracture of the conductive film were observed during bending, twisting, and even folding.

The electrothermal actuation is based on the CHE and CTE of each active layer. After applying voltage to the actuator, the generated electrical current is converted into thermal energy via Joule heating, rapidly heating the conductive film. The produced thermal energy is then transferred to paper and PP layers, causing a substantial hygroscopic contraction of paper upon its moisture desorption. Since the PP film is inert to change in humidity, the bilayer actuator bends toward the paper side. Meanwhile, significant CTE mismatch between paper and PP film induces larger thermal expansion in the PP film and further accelerates the bending deformation of the actuator. Once the applied voltage is turned off, the actuator approximately returns to its original shape due to the heat loss followed by its moisture absorption from the environment. This dual‐mode actuation mechanism has led to large bending deformations of such electrically activated actuators (Figure 1d).

When an actuator is heated, change in the electrical resistance of the embedded conductive film is governed by its inherent thermoresistivity (i.e., temperature dependence of the resistance), as well as its piezoresistive response to the induced mechanical strain during bending deformation of the actuator. Thus, the overall relative resistance change of the conductive film can be expressed as

(1) $${\left(\frac{\Delta R}{R_{0}}\right)}_{\mathrm{Total}}=\alpha_{T}.\Delta T+\mathrm{GF}.\varepsilon$$

where ∆*R* is the resistance change, *R*_0_ is the initial resistance of the conductive film, *α_T_* is the TCR of the conductive film, ∆*T* is the temperature change of the actuator, GF is the gauge factor or strain sensitivity of the conductive film, and ε is the bending strain accommodated by the conductive film. According to this equation, the temperature‐dependent resistance change of the actuator interferes with its piezoresistive response. Consequently, the real ε cannot be measured from the total resistance change of the conductive film since it differs under the same strain but at different temperatures. To avoid this unwanted crosstalk, *α_T_* of the conductive film should be very small (nearly zero) to remove the correlation between temperature and resistance. Such a conductive film enables fully decoupled Joule heating and temperature‐independent strain sensing capabilities, thereby yielding an integrated self‐sensing actuator. From the equation, high GF of the conductive film would also permit sensitive and precise monitoring of the actuator's deformation.

In the search for functional materials that can fulfill the aforementioned requirements, we considered hybrid films of graphite and CNTs due to their several key features that could influence the self‐sensing performance of actuators (see “Materials Selection Criteria” section in the Supporting Information for details). First, metallic graphite and semiconducting CNT films possess positive and negative TCR values, respectively.^32^ Considering their opposite change in the electrical resistance upon temperature alteration, one can obtain a hybrid film with negligible TCR value. This can be attained by optimizing the mass ratio of graphite microparticles and CNTs within the percolation network, as demonstrated by Luo et al.^32^ Second, the high GF of the graphite film compensates the poor piezoresistivity of the CNT film in the hybrid configuration, allowing highly sensitive strain sensation of self‐sensing actuators.^26^, ^30^, ^31^, ^32^, ^33^, ^34^ Third, the hybridization process enhances the electrical conductivity through interface between graphite microparticles and CNTs, which in turn reduces the driving voltage and energy consumption of actuators.^32^, ^35^, ^36^, ^37^

We initially evaluated the thermoresistive behavior of graphite, CNT, and different hybrid films by varying the mass ratio of CNTs to graphite microparticles. We used glass as the supporting substrate given its low thermal expansion (CTE_Glass_ ≈ 9 ppm K^−1^), which holds coated conductive films almost intact during temperature variations. The resistance of each sample was then collected when a temperature difference was added to the glass substrate (see the Experimental Section for more details). **Figure** **2**a shows relative change of the resistance as a function of temperature. As expected, neat graphite films exhibited positive TCR behavior due to their enhanced phonon/charge carrier scattering and subsequent reduction in the carrier mobility with increasing their temperature. By contrast, negative TCR of CNT films is because of the variable‐range hopping mechanism, which facilitates the charge carrier mobility at higher temperatures.^32^ When the temperature of graphite and CNT films was increased from 40 to 100 °C, their relative resistance changes were 7.05% ± 0.47% and −1.30% ± 0.24%, respectively. The thermoresistivity of conductive films made of hybrid inks with mass ratios of 0.08 (hybrid 1), 0.1 (hybrid 2), and 0.12 (hybrid 3) are also provided in Figure 2a. The hybridization process significantly diminished the temperature dependency of the resistance. As depicted in the figure, the resistance of hybrid 3 films tends to become constant regardless of their temperature. The relative resistance changes of hybrid 1, hybrid 2, and hybrid 3 films at 100 °C (with respect to the resistance at 40 °C) were 1.74% ± 0.31%, 0.72% ± 0.35%, and 0.33% ± 0.20%, respectively. For each sample type, we then calculated the root‐mean‐square deviation (RMSD_Thermal_) of the relative resistance change. The respective RMSD_Thermal_ of graphite, CNT, hybrid 1, hybrid 2, and hybrid 3 films was 3.58%, 0.85%, 0.82%, 0.32%, and 0.18%, indicating significant temperature self‐compensation of hybrid 3 films. Similar behavior has been observed in poly(3,4‐ethylenedioxythiophene):poly(styrene sulfonate) (PEDOT:PSS) aerogels and hybrid microstructures.^32^, ^38^, ^39^ For example, a temperature self‐compensated pressure sensor made of dimethylsulfoxide treated PEDO:PSS aerogel could precisely read the pressure level at different temperatures (20–45 °C).^38^ Compared to previous reports, our strategy is easy‐to‐fabricate, scalable, cost‐effective, and environmentally friendly, accompanied by a broader temperature self‐compensation range.

**Figure 2:**
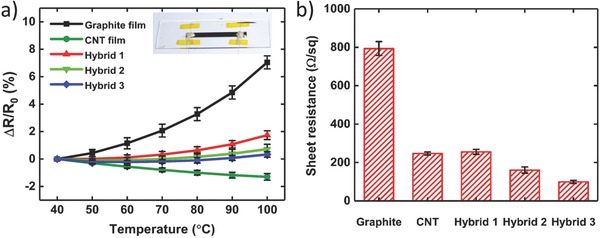
a) Relative resistance change of graphite, CNTs, and hybrid films coated on the glass substrate upon increasing the temperature. Inset: photograph of a hybrid 3 film with lateral dimensions of 40 mm × 4 mm (length × width) deposited on a glass microscope slide (*N* = 3, where *N* is the number of randomly tested samples). b) Sheet resistance of graphite, CNTs, and hybrid films deposited on paper (*N* = 5).

Figure 2b depicts the sheet resistance of graphite, CNT, and hybrid films. Rectangular shaped films were patterned on paper and their sheet resistances were calculated from *R*_S_ = *RW*/*L*, where *R* is the electrical resistance of conductive films measured from their two ends, *W* is the width (3 mm), and *L* is the length (30 mm) of conductive films. The sheet resistances of graphite and CNT films were 771 ± 69 and 247 ± 8 Ω sq^−1^, respectively, showing poor conductivity of graphite films on paper. It is evident in the figure that the sheet resistance of hybrid films decreased with increase in the CNTs content and reached to the lowest value of 99 ± 9 Ω sq^−1^ for hybrid 3 films. The conductivity enhancement of hybrid films is attributed to the good bonding and mutual interaction between graphite microparticles and CNTs. The SEM image on the surface of a neat graphite film coated on paper revealed interstitial space among many particles that prevents effective charge transport in the percolation network. These gaps were no longer visible in a hybrid 3 film by SEM observation, which is resulted from the infiltration of CNTs into the microvoids (Figure S5, Supporting Information). Moreover, CNTs would act as interconnects among tightly spaced graphite particles and thus promote the carrier mobility and conductivity.^32^, ^35^, ^36^, ^37^

**Figure** **3** characterizes the strain‐sensing performance of graphite, CNT, and hybrid films deposited on the paper–PP bilayer (see the Experimental Section for more information). All samples with lateral dimensions of 50 mm × 4 mm (length × width) were subjected to outward and inward bending cycles, while their electrical resistance was continuously recorded (Figure 3a). Figure 3b illustrates their electromechanical response for five bending‐straightening cycles. At each cycle, the chord length decreased from 50 mm to 25 mm and then returned to the original length with the speed of 8.90 mm s^−1^. The resistance of all samples increased under tension (outward bending) and dropped upon compressive strain (inward bending). Furthermore, their output signals were highly reversible under both tensile and compressive loading cycles. The piezoresistive mechanism can be explained by the connection–disconnection of overlapped materials.^26^, ^31^, ^34^ When the bilayer is bent outward, induced stretching in conductive films reduces the overlapped area and junctions of connected graphite particles and CNTs, and thus increases the resistance. Conversely, compressive bending cycle brings graphite particles and CNTs closer and increases their overlapped area, consequently, lowers the resistance.

**Figure 3:**
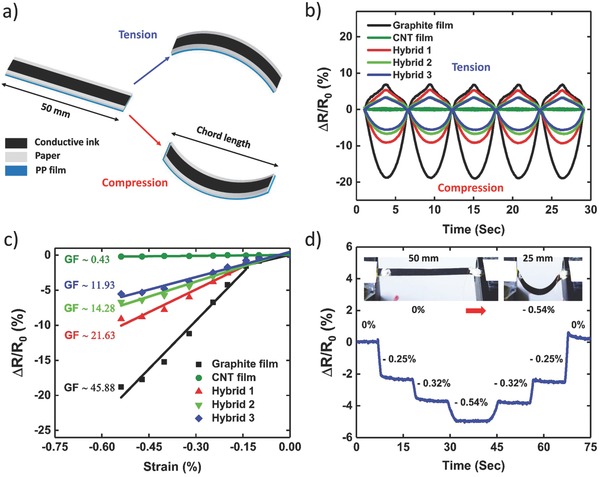
Strain sensing characterization of graphite, CNT, and hybrid films coated on the paper–PP bilayer. a) Schematic illustration of tension and compression tests for the piezoresistive evaluation of graphite, CNT, and hybrid films. b) Resistive response of graphite, CNT, and hybrid films to cyclic tension and compression. c) Relative change of the resistance versus compressive strain for graphite, CNT, and hybrid films. d) Piezoresistivity of a hybrid 3 film to stepwise loading cycles. Insets: photographs of the sample at straight and bent states.

Next, we quantitatively analyzed the magnitude of piezoresistivity by deriving the bending strain from ε = ±*h*/*r*, where *h* is the distance of conductive films from the neutral axis of the bilayer structure (≈71 µm) and *r* is the radius of curvature (see “Bending Strain” section and Figures S6 and S7 in the Supporting Information).^26^ The *r* value has a relationship with the chord length *c* as: *c* = 2*r* sin(*l*/2*r*), where *l* is the arc length of samples (50 mm). Since conductive films are compressed during bending deformation of actuators, we only plotted relative change of the resistance versus compressive strain in Figure 3c. The GFs are then calculated from the slope of the curves (GF = ∆*R*/*R*_0_ε). The GFs of hybrid 2 and hybrid 3 films are 11.93 and 14.28, respectively, coupled with a highly linear piezoresistive response (*R* ^2^ ≥ 0.95). Although graphite and hybrid 1 films possess higher GFs, their response is highly nonlinear. On the other hand, GF of the CNT film is very low (0.43) and unsuitable for sensitive strain sensing applications.

In order to assess the effect of the temperature self‐compensation of hybrid films on the self‐sensation of actuators, we defined the SNR as

(2) $$\mathrm{SNR}=\frac{\mathrm{GF}}{{\mathrm{RMSD}}_{\mathrm{Thermal}}}$$

Notably, the SNR of hybrid 3 films (66.28) is 5.17 and 129.96 times greater than that of graphite (12.82) and CNT (0.51) films, respectively. Significant improvement in the SNR of hybrid 3 films is due to their excellent temperature‐independent sensing capability. The SNRs of hybrid 2 (26.38) and hybrid 3 (44.62) films are still much higher than the SNRs of neat counterparts, which prove the superiority of the hybridization strategy in the self‐sensing performance of soft actuators. The enhanced SNR not only provides accurate sensing information but also makes the signal reading easier. Considering the higher SNR of hybrid 3 films together with their lower sheet resistance, the hybrid 3 conductive ink was utilized to prepare paper‐based self‐sensing actuators.

Additional tests were conducted to further investigate the strain sensing and electrothermal performance of hybrid 3 films coated on the paper–PP bilayer. Figure 3d depicts the resistive response of a sample to a series of step‐hold (holding time ≈ 10 s) loading cycles. It could detect various strain levels (i.e., −0.25%, −0.32%, and −0.54%) with apparent resistance shift and no obvious overshoot. The sample reasonably persevered its piezoresistivity for more than 1000 repeated bending–straightening cycles, illustrating high‐performance strain sensing properties of hybrid 3 films (Figure S8, Supporting Information). In addition, the temperature of the Joule‐heated hybrid 3 films appeared linearly proportional to the input electric power (Figure S9, Supporting Information). Under the input power of 215 mW cm^−2^, the maximum surface temperature of a hybrid 3 film reached over 103 °C within 23 s (Figure S10, Supporting Information). The temperature morphology was uniform throughout the heating area, which is important for efficient heat transfer to actuating materials. For further information on energy efficiency, output force generation, long‐term stability, and actuation speed of our actuators, we refer the reader to our previous work.^1^

**Figure** **4**a demonstrates the self‐sensing capability of hybrid 3 films‐based actuators. Through the conductive layer of actuators, we applied voltage and measured the electrical resistance at the same time. When 30 V was applied to an actuator, the conductive film immediately responded to mechanical actuation. Its resistance gradually decreased as the accommodated compressive strain increased (see Movie S1, Supporting Information). As shown in the figure, relative change of the resistance matches well with the actuator tip displacement. For example, the self‐sensing actuator exhibited −1.40% resistance change for around 13.9 mm tip displacement. Accordingly, decreasing the applied voltage resulted in a smaller resistance change due to less deformation of the actuator (see Figure S11, Supporting Information). Moreover, the resistance change of the actuator was reversible for several on/off electric power cycles. Thus, unlike previous integrated actuators, the dynamic motion of our actuators can be monitored merely through two input electric terminals, without any need for additional sensing components or input energies.

**Figure 4:**
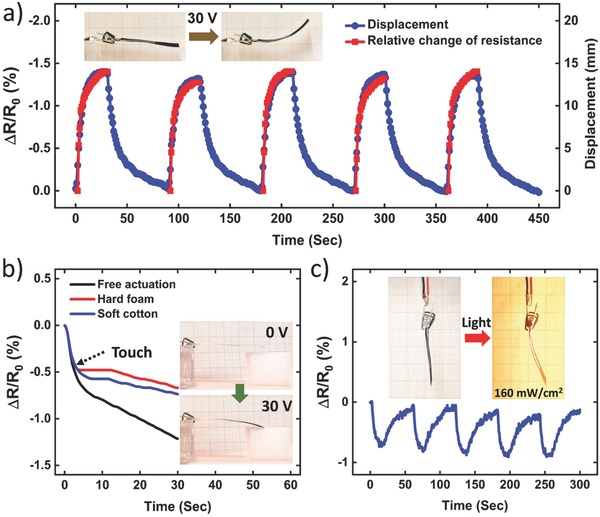
a) Self‐sensing function of a paper actuator; insets, displacement of the actuator before and after application of 30 V. b) Sensory response of an actuator during free actuation and touching hard and soft objects; insets, photographs of the actuator when touching a hard polymer foam. c) Piezoresistive response of an actuator to on‐off light switching. Insets: photographs of the actuator before and after light irradiation for 15 s.

As a control experiment, we also examined the sensory response of constrained actuators (actuators with zero deformation). One actuator was fixed on a flat glass substrate (straight actuator) and another actuator was wrapped around a plastic tube (bent actuator), and their resistance changes were recorded during electrothermal excitation (see Figure S12 and Movie S2, Supporting Information). The resistance of actuators stayed nearly constant (deviation <0.08%) under both straight and bending conditions though their temperature increased to around 100 °C, verifying that the resistance change of hybrid 3 films based actuators is directly associated with their bending state.

Along with bending deformation, our self‐sensing actuators can also detect the touch event. To demonstrate this ability, we measured the output signal of an actuator while touching a hard polymer foam and soft cotton (see Figure S13, Supporting Information). Figure 4b gives the resistance change of the actuator during free bending actuation as well as touching hard and soft objects. The sensory feedback of the actuator was identical upon touching, suggesting that the self‐sensing actuator can actively recognize touch in real time. As a normal response, the resistance of the actuator constantly decreased for free bending deformation. By contrast, the signal stabilized and remained constant once the actuator tip touched the surface of objects. Then, the resistance slowly decreased due to the further body deformation of the actuator (see Figure S14 and Movie S3, Supporting Information). The feedback response was similar for both hard and soft materials. However, the actuator exhibited more resistance shift when touching soft cotton due to the deformation of cotton that induces larger tip displacement. These results clearly demonstrate that our self‐sensing actuators could not only track the actuation displacement but also could distinguish the touch state.

Not limited to electricity, paper‐based actuators also respond to light irradiation and relative humidity (RH) changes, as we demonstrated in our previous work.^1^ To show the self‐sensing capability of hybrid 3 based actuators to these external stimuli, an actuator was placed in front of a light source (a 100 W lamp) at a distance of around 8 cm. When the light was projected to the surface of the actuator (light intensity of around 160 mW cm^−2^), the hybrid film quickly converted the photonic energy to the thermal energy due to the optical absorption of both graphite and CNTs.^7^, ^12^ The actuator tip showed 7 mm displacement in 15 s (see inset of Figure 4c). The infrared (IR) thermal image on the surface of the actuator revealed around 24 °C increases in the temperature of the hybrid film upon irradiation (see Figure S15, Supporting Information). Figure 4c gives the real‐time piezoresistive response of the actuator to on–off light cycles. The resistance decreased during the light exposure due to the bending deformation of the actuator, while recovered to its original value when the light source was switched off. Besides, hybrid 3 films retained their great temperature self‐compensation under different RH levels (Figure S16, Supporting Information). Therefore, the RH‐induced shape‐changing of our actuators can also be monitored via integrated strain sensors.

In summary, we leveraged the basic physical properties of functional materials to develop high‐performance and integrated self‐sensing actuators. The high CHE of the copy paper suitably complemented the high CTE of the PP film for large bending deformation of bilayer actuators. The hybrid films of graphite microparticles and CNTs deposited on paper eliminated the dependency on image processing or other sensing components for motion detection and allowed real‐time sensing capabilities via only a pair of input electric terminals. The self‐sensing actuation of paper‐based actuators can still be improved by reducing the resistance of hybrid films, tailoring the stiffness of the bilayer structure, and changing the porosity and fiber orientation of paper. We anticipate that our material approach will provide new insights in the design of integrated intelligent systems with synergistic multiple functions.

## Experimental Section

*Ink Preparation*: The conductive inks were prepared according the procedures described in a previous paper with some modifications.^40^ The mixture of 0.25 g ethyl cellulose (EC) (viscosity of 22 cP, Sigma‐Aldrich), 2.5 mL terpineol (mixture of isomers, anhydrous, Sigma‐Aldrich), and 7.5 mL isopropyl alcohol (IPA) was added to a glass bottle and magnetically stirred (speed: 1700 rpm) at 70 °C for 15 min. When EC was fully dispersed, 0.5 g graphite microparticles (particle size <20 µm, Sigma‐Aldrich) was added to the solution followed by further stirring under the same speed and temperature for 30 min. Then, the open capped bottle was stirred at 150 °C for 90 min to fully evaporate the IPA. The mixture was cooled down to the room temperature and the resultant viscous graphite conductive ink was kept for further experiments. The CNT (multiwalled CNTs with length (diameter) of 3–6 µm (10 nm), Sigma‐Aldrich) and hybrid conductive inks were prepared with the same procedures. Instead of graphite, 50 mg of CNTs was added to the mixture of EC, terpineol, and IPA to prepare the CNT conductive ink. The hybrid conductive inks were prepared by varying the mass ratio of CNTs to graphite microparticles. In order to tune the content of graphite microparticles and CNTs in hybrid inks, a fixed amount of graphite (0.25 g) was hybridized by CNTs with masses of 20, 25, and 30 mg.

*Fabrication of Paper‐Based Actuators*: First, U‐shaped heating circuits with lateral dimensions of 35–45 mm × 10 mm (length × width) were patterned on an A4 copy paper (80 g m^−2^, 110 µm thick, Inapa Tecno) by a 110 µm thick Kapton tape. The conductive ink made of graphite and CNTs was uniformly screen printed on the patterned paper by a steel blade. After removing Kapton tape, the excess paper was trimmed away and the deposited viscous ink was cured at 100 °C for 30 min. The solidified pattern was further annealed at 150 °C for 30 min. Finally, the PP self‐adhesive film (40 µm thick, Tesafilm transparent) was attached to the backside of the patterned paper to form a bilayer structure. The electrical connection of actuators in the fixed ends was easily established using small flat alligator clips. The displacement of actuators was analyzed by taking sequential optical images and postprocessing. It is noted that obvious bending actuations were observed only when actuators were patterned in the width direction of the copy paper. On the contrary, there was no bending movement once actuators were fabricated along the length direction of the paper. This behavior is attributed to the high anisotropic hygroscopic properties of the copy paper.^1^

*Characterization of the Temperature Self‐Compensation*: Graphite, CNT, and hybrid films with respective length and width of 40 and 4 mm were deposited on the glass supporting substrate. After ink curing at 100 °C for 30 min, copper wires were connected to two ends of each film using silver conductive epoxy (silver loaded epoxy adhesives, RS Components). All samples were put onto a hotplate (MSH‐20D, WiseStir) under 150 °C for another 30 min to anneal the conductive patterns and cure the silver paste. Right after, the temperature of samples was increased stepwise from 40 to 100 °C (10 °C for each step). Samples at each step were held for 10 min to make sure that the temperature profile was fully stabilized and then measured their electrical resistance by an LCR meter (ST2822A, SourceTronic). It is noteworthy to mention that the annealing process is important to remove residual organic components and decompose the EC polymer stabilizer for conductivity enhancement.^40^

*Strain‐Sensing Characterization*: Rectangular shaped graphite, CNT, and hybrid films (length of 50 mm × width of 4 mm) were deposited on the paper–PP film bilayer. Copper wires were attached to two ends of each sample by silver conductive epoxy (cured at 100 °C for 2 h). The strain sensing tests were then conducted by clamping the samples on a motorized moving stage (M‐605 High‐Accuracy Translation Stage, Physik Instrumente) and measuring their electrical resistance with data acquisition (DAQ) system (USB X Series Multifunctional DAQ, National Instruments) under different mechanical loading conditions.

*Microscopic Imaging*: The SEM images were taken by a scanning electron microscope (Supra 55VP, ZEISS). The 3D surface profile was measured by a 3D laser‐scanning microscope (VK‐X200 Series, KEYENCE).

*Surface Temperature Mapping*: An IR thermal camera (FLIR ONE) was used to capture the temperature profile of samples under different input electric powers, voltages, and light irradiation.

*Self‐Sensing Actuation*: To characterize the self‐sensing actuation of electrically activated actuators, the electrical current passing through conductive films was recorded by a digital multimeter (VC‐20, VOLTCRAFT). The signal was then converted to the relative resistance change. For light activated actuators, the real‐time resistance was measured by DAQ setup. The light intensity was measured by a thermal power sensor (S175C‐Microscope Slide Thermal Power Sensor, THORLABS). For Movies S1–S3 of the Supporting Information, sensing signals were collected as the current by the digital multimeter.

## Conflict of Interest

The authors declare no conflict of interest.

## Supporting information

Supplementary

Supplementary

Supplementary

Supplementary

## References

[1] M. Amjadi , M. Sitti , ACS Nano 2016, 10, 10202. 27744680 10.1021/acsnano.6b05545

[2] G. Z. Lum , Z. Ye , X. Dong , H. Marvi , O. Erin , W. Hu , M. Sitti , Proc. Natl. Acad. Sci. USA 2016, 113, 201608193. 10.1073/pnas.1608193113 PMC5068264 27671658

[3] L. Hines , K. Petersen , G. Z. Lum , M. Sitti , Adv. Mater. 2017, 29, 1603483. 10.1002/adma.201603483 28032926

[4] S. M. Mirvakili , I. W. Hunter , Adv. Mater. 2017, 29, 1604734.

[5] Y. Hu , J. Liu , L. Chang , L. Yang , A. Xu , K. Qi , P. Lu , G. Wu , W. Chen , Y. Wu , Adv. Funct. Mater. 2017, 27, 1704388.

[6] H.‐W. Huang , M. S. Sakar , A. J. Petruska , S. Pané , B. J. Nelson , Nat. Commun. 2016, 7, 12263. 27447088 10.1038/ncomms12263 PMC5512624

[7] Y. Tai , G. Lubineau , Z. Yang , Adv. Mater. 2016, 28, 4665. 27061392 10.1002/adma.201600211

[8] Q. Li , C. Liu , Y.‐H. Lin , L. Liu , K. Jiang , S. Fan , ACS Nano 2015, 9, 409. 25559661 10.1021/nn505535k

[9] M. M. Hamedi , V. E. Campbell , P. Rothemund , F. Güder , D. C. Christodouleas , J. F. Bloch , G. M. Whitesides , Adv. Funct. Mater. 2016, 26, 2446.

[10] S. Taccola , F. Greco , E. Sinibaldi , A. Mondini , B. Mazzolai , V. Mattoli , Adv. Mater. 2015, 27, 1668. 25556552 10.1002/adma.201404772 PMC4369129

[11] H.‐P. Phan , T. Dinh , T.‐K. Nguyen , A. Vatani , A. R. Md Foisal , A. Qamar , A. R. Kermany , D. V. Dao , N.‐T. Nguyen , Appl. Phys. Lett. 2017, 110, 144101.

[12] M. Weng , P. Zhou , L. Chen , L. Zhang , W. Zhang , Z. Huang , C. Liu , S. Fan , Adv. Funct. Mater. 2016, 26, 7244.

[13] L. Hines , K. Petersen , M. Sitti , Adv. Mater. 2016, 28, 3690. 27008455 10.1002/adma.201600107

[14] J. Shintake , S. Rosset , B. Schubert , D. Floreano , H. Shea , Adv. Mater. 2016, 28, 231. 26551665 10.1002/adma.201504264

[15] M. Duduta , R. J. Wood , D. R. Clarke , Adv. Mater. 2016, 28, 8058. 27376638 10.1002/adma.201601842

[16] S. Palagi , A. G. Mark , S. Y. Reigh , K. Melde , T. Qiu , H. Zeng , C. Parmeggiani , D. Martella , A. Sanchez‐Castillo , N. Kapernaum , F. Giesselmann , D. S. Wiersma , E. Lauga , P. Fischer , Nat. Mater. 2016, 15, 647. 26878315 10.1038/nmat4569

[17] H. Shahsavan , S. M. Salili , A. Jákli , B. Zhao , Adv. Mater. 2017, 29, 1604021. 10.1002/adma.201604021 27859776

[18] H. Shahsavan , S. M. Salili , A. Jákli , B. Zhao , Adv. Mater. 2015, 27, 6828. 26418411 10.1002/adma.201503203

[19] H. Zeng , O. M. Wani , P. Wasylczyk , R. Kaczmarek , A. Priimagi , Adv. Mater. 2017, 29, 1701814. 10.1002/adma.201701814 28589679

[20] H. Cheng , F. Zhao , J. Xue , G. Shi , L. Jiang , L. Qu , ACS Nano 2016, 10, 9529. 10.1021/acsnano.6b04769 27636903

[21] H. Arazoe , D. Miyajima , K. Akaike , F. Araoka , E. Sato , T. Hikima , M. Kawamoto , T. Aida , Nat. Mater. 2016, 15, 1084. 27429210 10.1038/nmat4693

[22] J. Gong , H. Lin , J. W. Dunlop , J. Yuan , Adv. Mater. 2017, 29, 1605103. 10.1002/adma.201605103 28218811

[23] Y. Hu , G. Wu , T. Lan , J. Zhao , Y. Liu , W. Chen , Adv. Mater. 2015, 27, 7867. 26498737 10.1002/adma.201502777

[24] M. Vural , Y. Lei , A. Pena‐Francesch , H. Jung , B. Allen , M. Terrones , M. C. Demirel , Carbon 2017, 118, 404.

[25] Z. Ji , C. Yan , B. Yu , X. Wang , F. Zhou , Adv. Mater. Interfaces 2017, 4, 1700629.

[26] M. Amjadi , M. Turan , C. P. Clementson , M. Sitti , ACS Appl. Mater. Interfaces 2016, 8, 5618. 26842553 10.1021/acsami.5b12588

[27] J. C. Yeo , H. K. Yap , W. Xi , Z. Wang , C. H. Yeow , C. T. Lim , Adv. Mater. Technol. 2016, 1, 1600018.

[28] T. Helps , J. Rossiter , Soft Rob. 2018, 5, 175. 10.1089/soro.2017.0012 PMC5905876 29211627

[29] H. Zhao , K. O'Brien , S. Li , R. F. Shepherd , Sci. Rob. 2016, 1, eaai7529.

[30] L. Hu , J. W. Choi , Y. Yang , S. Jeong , F. La Mantia , L.‐F. Cui , Y. Cui , Proc. Natl. Acad. Sci. USA 2009, 106, 21490. 19995965 10.1073/pnas.0908858106 PMC2799859

[31] X. Liao , Q. Liao , X. Yan , Q. Liang , H. Si , M. Li , H. Wu , S. Cao , Y. Zhang , Adv. Funct. Mater. 2015, 25, 2395.

[32] S. Luo , T. Liu , Adv. Mater. 2013, 25, 5650. 23939948 10.1002/adma.201301796

[33] M. Amjadi , Y. J. Yoon , I. Park , Nanotechnology 2015, 26, 375501. 26303117 10.1088/0957-4484/26/37/375501

[34] M. Amjadi , K. U. Kyung , I. Park , M. Sitti , Adv. Funct. Mater. 2016, 26, 1678.

[35] U. Khan , I. O'Connor , Y. K. Gun'ko , J. N. Coleman , Carbon 2010, 48, 2825.

[36] A. Benchirouf , C. Müller , O. Kanoun , Nanoscale Res. Lett. 2016, 11, 4. 26732277 10.1186/s11671-015-1216-5 PMC4701710

[37] D. Cai , M. Song , C. Xu , Adv. Mater. 2008, 20, 1706.

[38] S. Han , F. Jiao , Z. U. Khan , J. Edberg , S. Fabiano , X. Crispin , Adv. Funct. Mater. 2017, 27, 1703549.

[39] W. He , G. Li , S. Zhang , Y. Wei , J. Wang , Q. Li , X. Zhang , ACS Nano 2015, 9, 4244. 25811954 10.1021/acsnano.5b00626

[40] W. J. Hyun , E. B. Secor , G. A. Rojas , M. C. Hersam , L. F. Francis , C. D. Frisbie , Adv. Mater. 2015, 27, 7058. 26439306 10.1002/adma.201503478

