# Supplementary material for: Self‐Sensing Paper Actuators Based on Graphite–Carbon Nanotube Hybrid Films
Source: Adv Sci (Weinh). 2018 May 16;5(7):1800239. doi: 10.1002/advs.201800239 (PMC6051221; doi:10.1002/advs.201800239)
Supplement: Supplementary file 1 — Supplementary [file ADVS-5-1800239-s001.pdf]

Copyright WILEY-VCH Verlag GmbH & Co. KGaA, 69469 Weinheim, Germany, 2018.

ADVANCED SCIENCE
Open Access

Supporting Information

for *Adv. Sci.,* DOI: 10.1002/advs.201800239

Self-Sensing Paper Actuators Based on Graphite–Carbon Nanotube Hybrid Films

*Morteza Amjadi and Metin Sitti**

Copyright WILEY-VCH Verlag GmbH & Co. KGaA, 69469 Weinheim, Germany, 2018.

Supporting Information

Self-Sensing Paper Actuators based on Graphite-Carbon Nanotube Hybrid Films

*Morteza Amjadi and Metin Sitti**

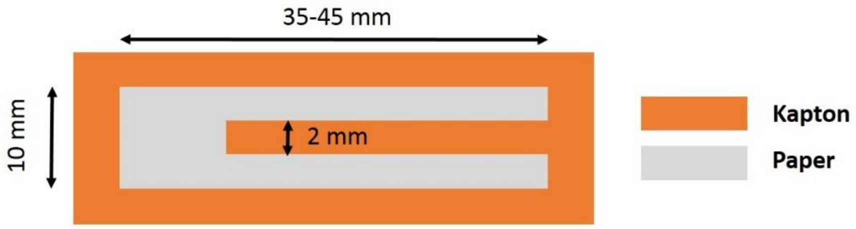

Figure S1: Dimensions of the paper substrate patterned with Kapton tape.

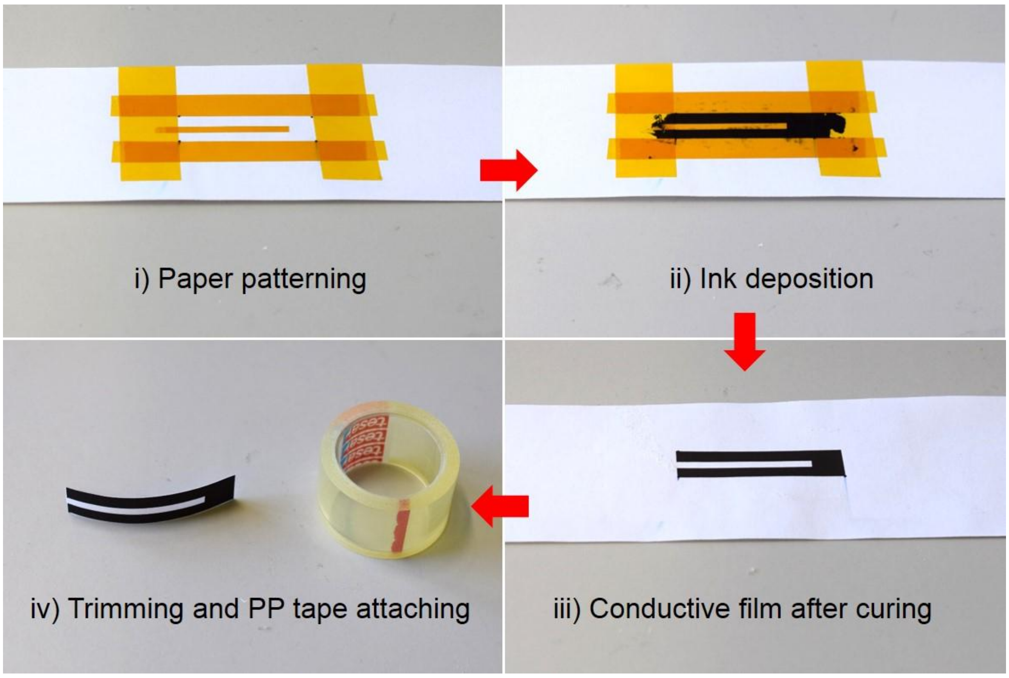

Figure S2: Photographs of the fabrication steps of the actuator.

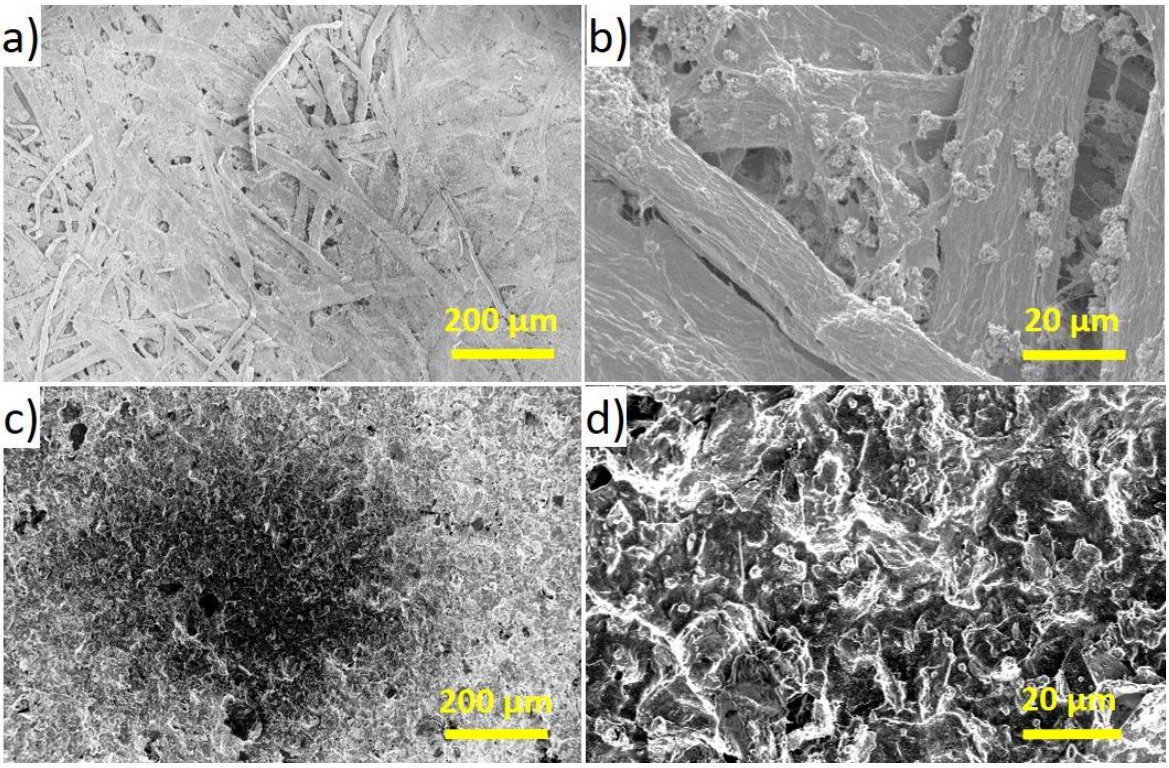

Figure S3: a) SEM image on the surface of the bare paper. b) Magnified SEM image on the surface of the bare paper. c) SEM image on the surface of the conductive ink coated paper. d) Magnified SEM image on the surface of the conductive ink coated paper.

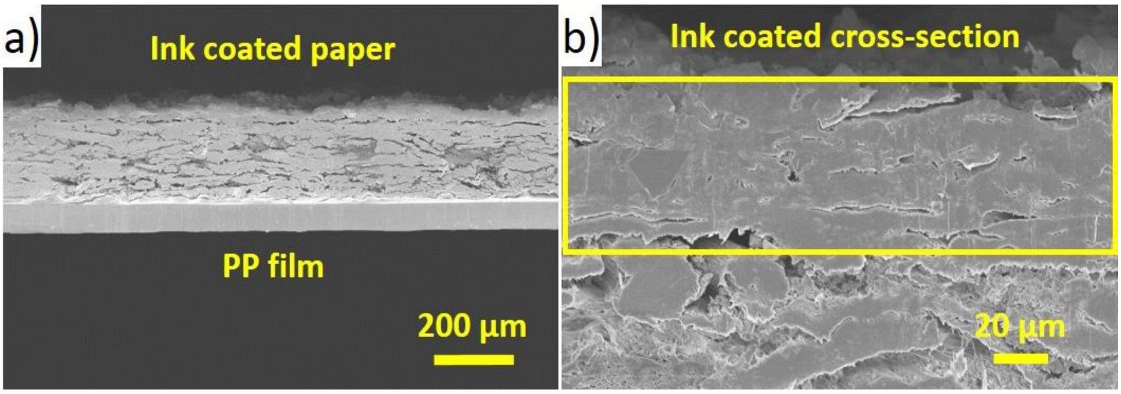

Figure S4: a) Cross-sectional SEM image of the actuator. b) Cross-sectional SEM image of the paper at the conductive layer coated region, showing infiltration of the conductive ink and subsequent binding between conductive film and paper.

Materials Selection Criteria: In our design, the conductive film coated on paper has to fulfill following minimum requirements for developing high-performance self-sensing actuators. First, its electrical conductivity should be high enough for low power and voltage operation of actuators. Second, besides the high conductivity, the conductive layer must possess sufficient strain-sensitivity or GF for accurate monitoring of the actuation deformation. Third, it must have very low TCR value so that strain sensitivity can be decoupled from the electroresistive behavior of the film during electrical stimulation. Forth, for successful patterning of the conductive film over paper, the functional material should be in the form of viscous ink due to the high porosity and hydrophilic nature of the paper substrate. Considering these basic requirements, we found that hybrid ink of graphite and CNTs can be potentially utilized. In principle, hybrid films made of any positive and negative TCR materials can be utilized for temperature self-compensation applications upon satisfying abovementioned criteria. Linear TCR behavior of both materials in a wide range of temperatures is also important for temperature self-compensation of the hybrid over a wide temperature range. Optimization process is necessary to find the best ratio of two materials for great self-compensation.

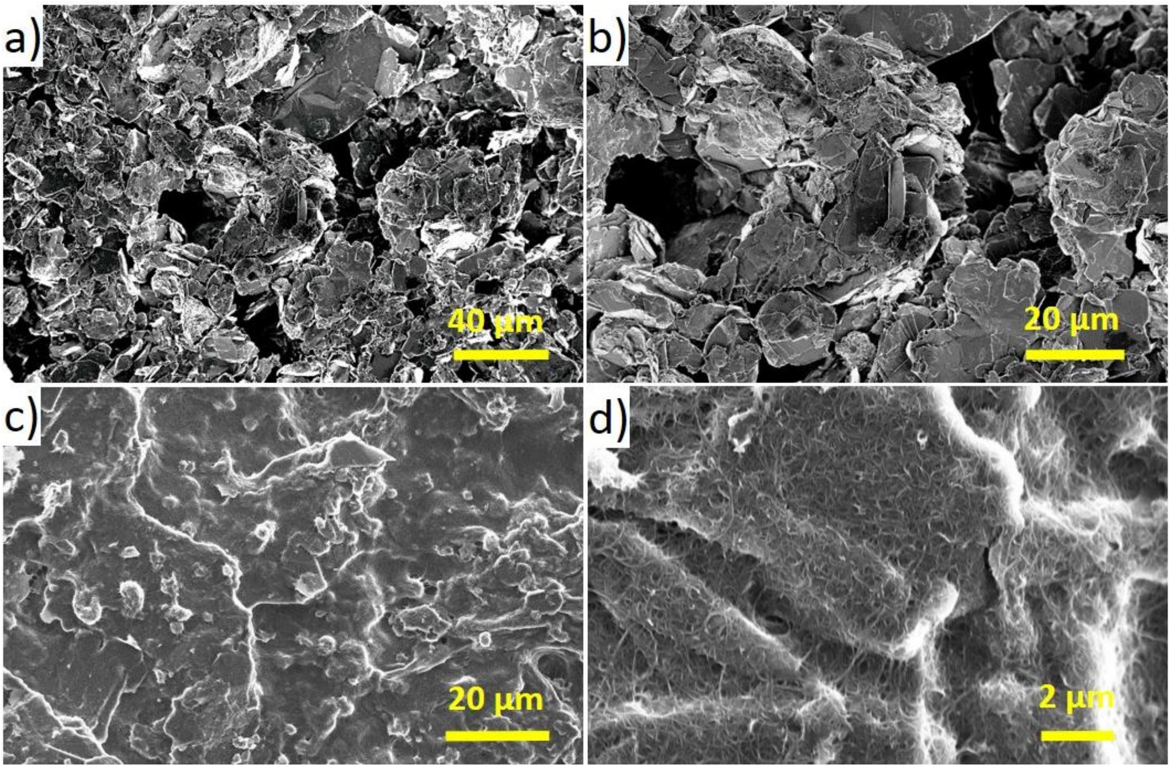

Figure S5: a) SEM image on the surface of a graphite film coated on paper. b) Magnified SEM image on the surface of the graphite film coated on paper. c) SEM image on the surface of a hybrid 3 film coated on paper. d) Magnified SEM image on the surface of the hybrid 3 film coated on paper, showing strong interaction between graphite particles and CNTs.

Bending strain:

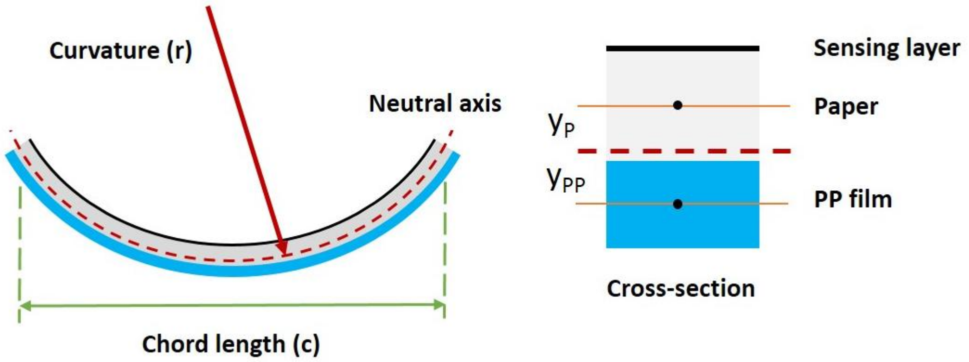

Figure S6: Schematic illustration of the cross-section of the conductive layer coated paper-PP film bilayer.

Figure S6 depicts the cross-section of the multilayered structure of paper-based actuators. To calculate the distance of the sensing layer from the neutral axis of the bilayer structure (h), we assumed that the thickness of the sensing layer is very small and not affects mechanical properties of paper. We confirmed this by taking a 3D laser-scanning microscopy image on the surface of the hybrid 3 film coated paper. As shown in Figure S7, there is no considerable height difference between conductive ink coated region and bare paper, proving that the conductive ink sufficiently penetrated into the porous structure of paper. The thickness of the paper substrate and PP film are 110 and 40 µm, respectively. Therefore,

$y_p+y_{pp} = 75\ \mu m$

where, y_p and y_{pp} are the distance of the centroid of paper and PP film from the neutral axis, respectively. The neutral axis is an axis in the cross-section where there is no bending stress over the bilayer. Thus,

$E_p y_p A_p - E_{pp} y_{pp} A_{pp} = 0$

where, E_p (E_{pp}) and A_p (A_{pp}) are the Young's modulus and cross-sectional area of the paper substrate (PP film), respectively. Assuming that E_p and E_{pp} are 2 and 1.5 GPa, respectively. Then,

$2\times y_p\times 110+1.5\times y_{pp}\times 40 = 0 \Rightarrow 3.67y_p = y_{pp} \Rightarrow y_p = 16.07\ \mu m$

Therefore,

h = 16.07 + 55 = 71.07 µm

The bending strain was then calculated from $\varepsilon = \pm h/r$, where r is the radius of curvature. r was derived from $c=2r\ sin(l/2r)$, where l is the arc length of the bilayer (50 mm).

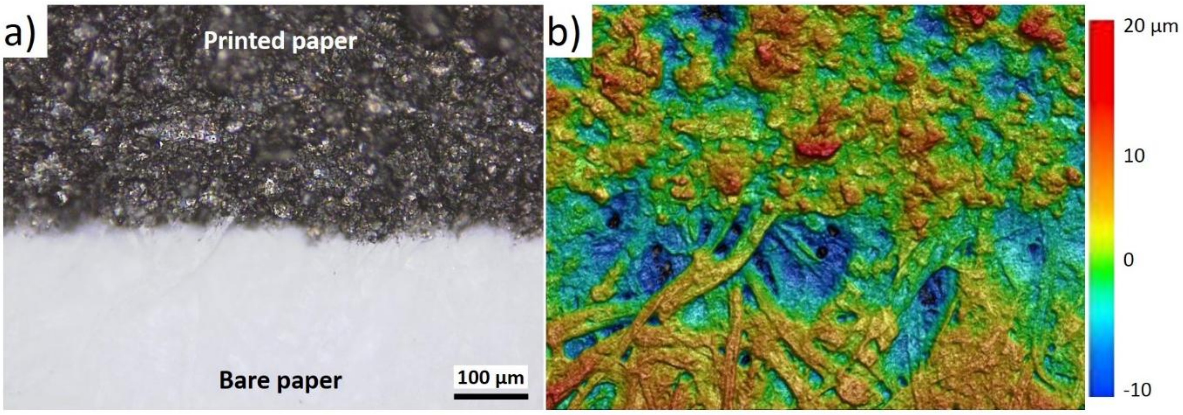

Figure S7: a) Optical microscopic image of the hybrid 3 film coated paper. b) Corresponding 3D laser-scanning microscopic image on the surface of the sample, showing that the conductive ink has penetrated into the paper porous structure, forming a nanocomposite structure.

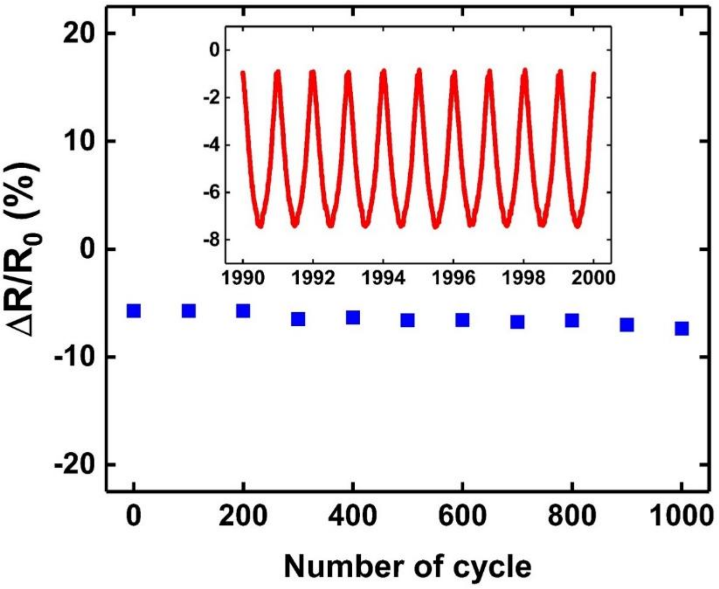

Figure S8: The relative resistance change of a hybrid 3 film coated paper-PP film bilayer for 1000 repeated bending-straightening cycles where the chord length decreased from 50 to 25

mm with the speed of 8.90 mm/s. Inset: electromechanical response of the sample in the last ten cycles.

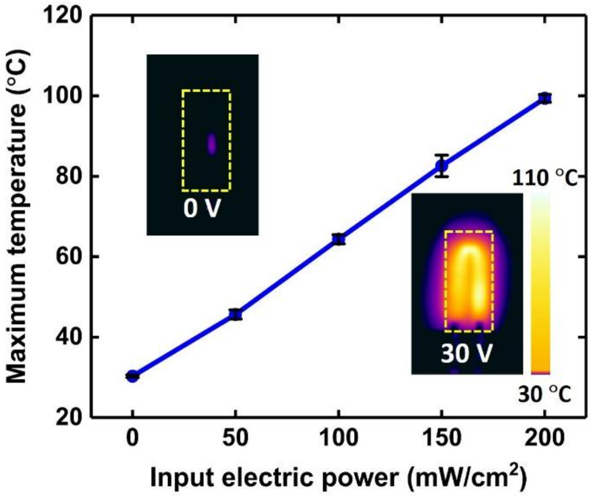

Figure S9: Maximum surface temperature of hybrid 3 paper heaters upon application of different input electric powers (N = 5). Insets: IR images on the surface of a paper heater before and after application of 30 V.

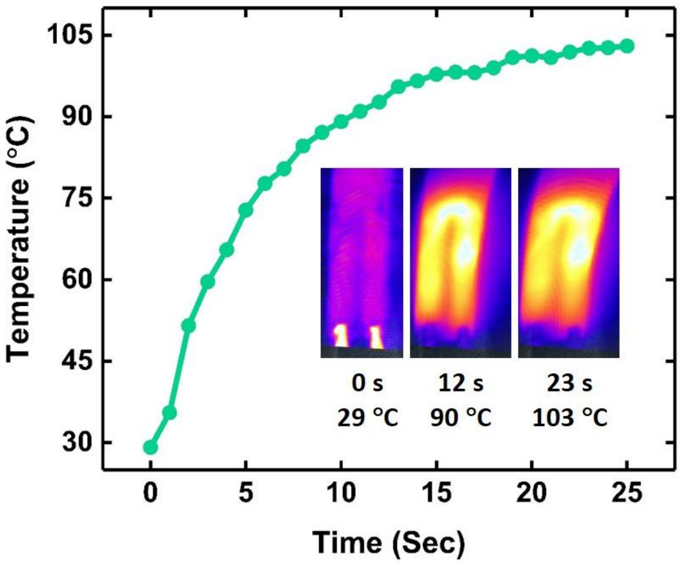

Figure S10: Temporal electrothermal response of a hybrid 3 film coated on paper-PP bilayer. Insets: sequential IR images on the surface of the paper heater.

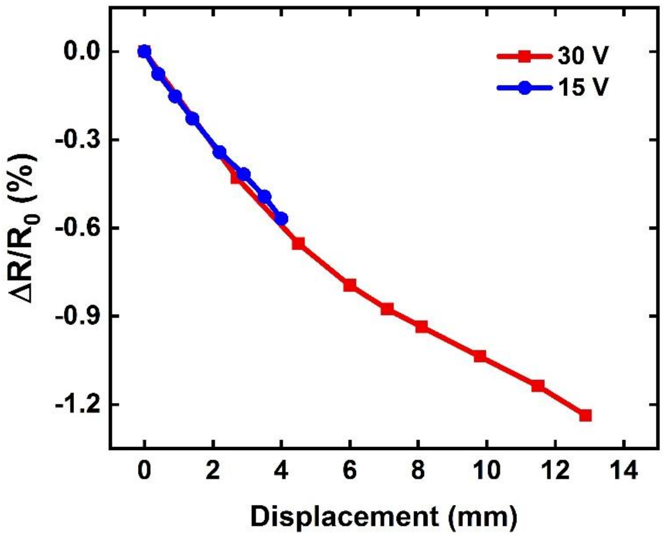

Figure S11: The relative resistance change versus the tip displacement for an actuator powered by 15 and 30 V.

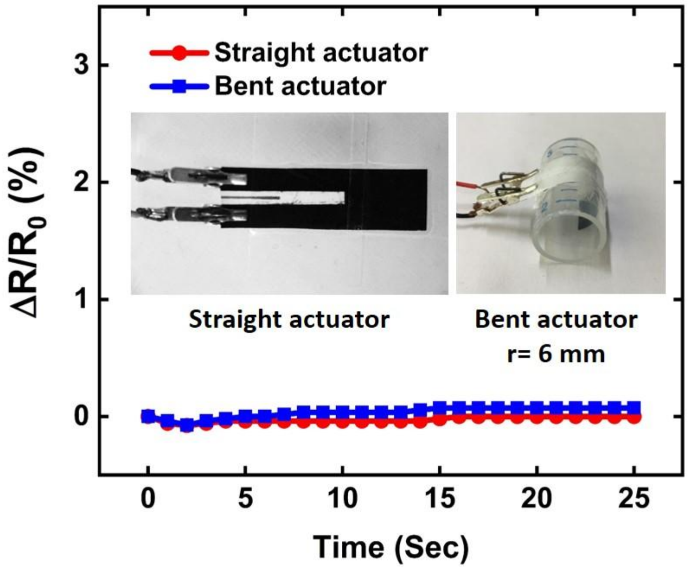

Figure S12: The relative resistance change of constrained actuators. Insets: photographs of the straight (left) and bent (right) constrained actuators.

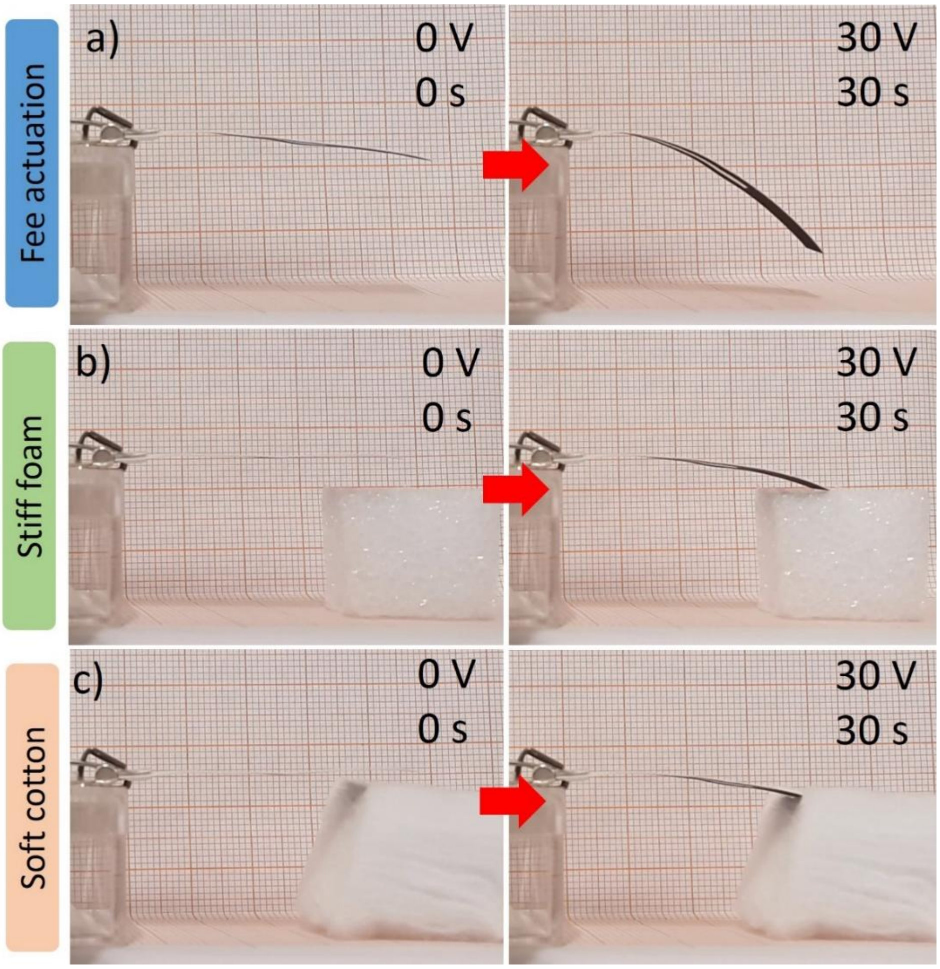

Figure S13: Photographs of an actuator upon a) free bending actuation, b) touching a hard polymeric foam, and c) touching soft cotton.

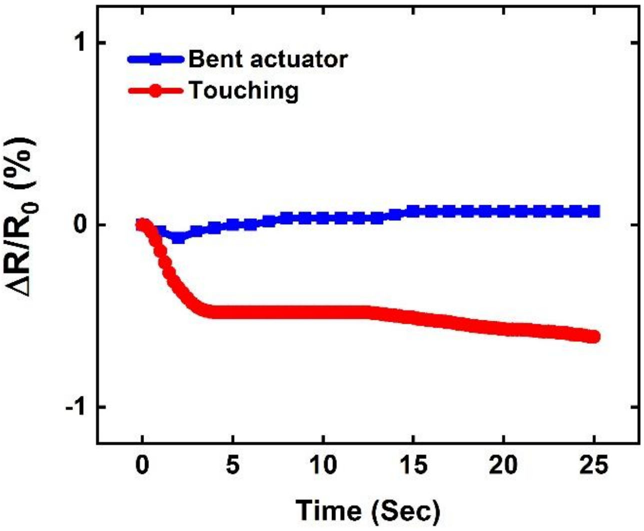

Figure S14: Response of actuator when wrapped around a tube with zero deformation (Bent actuator) and upon touching a hard polymeric foam, clearly showing the difference between two cases.

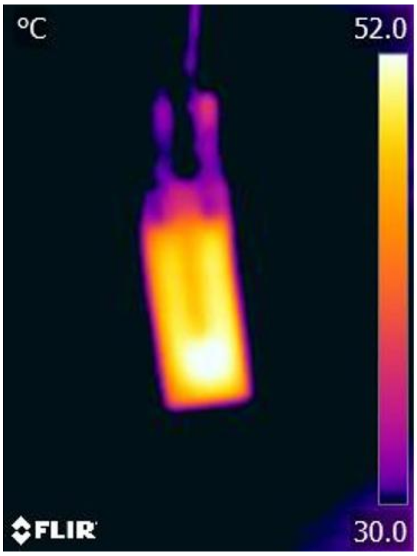

Figure S15: IR image on the surface of an actuator upon light irradiation.

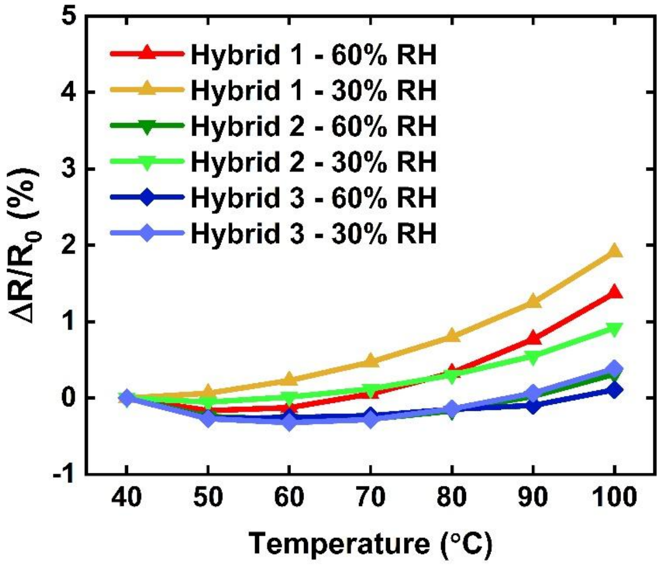

Figure S16: Thermoresistivity of hybrid films coated on the glass substrate under different RH levels.
